# Temperature Effect on the Compressive Behavior and Constitutive Model of Plain Hardened Concrete

**DOI:** 10.3390/ma13122801

**Published:** 2020-06-22

**Authors:** Ayman El-Zohairy, Hunter Hammontree, Eddie Oh, Perry Moler

**Affiliations:** Department of Engineering and Technology, Texas A&M University-Commerce, Commerce, TX 75429, USA; hhammontree@leomail.tamuc.edu (H.H.); Eddie.Oh@tamuc.edu (E.O.); Perry.Moler@tamuc.edu (P.M.)

**Keywords:** temperature, concrete, compressive strength, modulus of elasticity, constitutive model, mode of failure, strains, stresses

## Abstract

Concrete is one of the most common and versatile construction materials and has been used under a wide range of environmental conditions. Temperature is one of them, which significantly affects the performance of concrete, and therefore, a careful evaluation of the effect of temperature on concrete cannot be overemphasized. In this study, an overview of the temperature effect on the compressive behavior of plain hardened concrete is experimentally provided. Concrete cylinders were prepared, cured, and stored under different temperature conditions to be tested under compression. The stress–strain curve, mode of failure, compressive strength, ultimate strain, and modulus of elasticity of concrete were evaluated between the ages of 7 and 90 days. The experimental results were used to propose constitutive models to predict the mechanical properties of concrete under the effect of temperature. Moreover, previous constitutive models were examined to capture the stress–strain relationships of concrete under the effect of temperature. Based on the experimental data and the proposed models, concrete lost 10–20% of its original compressive strength when heated to 100 °C and 30–40% at 260 °C. The previous constitutive models for stress–strain relationships of concrete at normal temperatures can be used to capture these relationships under the effect of temperature by using the compressive strength, ultimate strain, and modulus of elasticity affected by temperature. The effect of temperature on the modulus of elasticity of concrete was considered in the ACI 318-14 equation by using the compressive strength affected by temperature and the results showed good agreement with the experimental data.

## 1. Introduction

Concrete is considered one of the most important materials in construction. Therefore, it is vital to explore the impact of engineering environments on concrete behavior. Factors affecting the concrete strength are temperature, water/cement (W/C) ratio, coarse/fine aggregate ratio, relative humidity, concrete age, and concrete curing [1]. Since concrete gains strength through a hydration process between cement and water curing, the temperature has a significant effect on the compressive behavior of concrete. The faster reaction is obtained at a higher temperature and subsequently higher strength. Whereas, that faster rate of hydration causes a reduction in the final strength of concrete because the physical form of the hardened cement paste is less well-structured and more porous at high temperatures [2]. 

Most of the mechanical properties of hardened concrete are also related to its compressive strength [3]. The uniaxial compression tests are commonly used to determine the strength and deformation properties of concrete. The standardized method for determining compressive strength is regulated in ASTM C387 [4]. Specimens are tested up to failure under the effect of uniaxial compression load. The compressive strength of concrete is calculated by dividing the recorded maximum load by the cross-sectional area [5].

Previous studies were performed on the compressive behavior of concrete at different temperatures. The compressive strength of concrete with the same moisture content decreased as the heating temperature increased [6,7]. The concrete curing temperatures below 5 °C or over 100 °C led to a reduction of almost 20% in the concrete strength [8]. The optimal mechanical performance of concrete was obtained when there was less difference between the ambient temperature and concrete temperature [9]. The setting time was affected by temperature [2]. The low-temperature setting time was as much as 195% of the setting time at 23 °C (73 °F). Whereas, the setting time was 68% at high temperatures. The strength of concrete had significant influences on the stress–strain responses both at room and elevated temperatures [10,11,12,13]. Because of the decrease in the compressive strength of concrete at high temperatures, the slope of the stress–strain curve decreased indicating reductions in the stiffness of concrete [10]. The strain corresponding to the peak strength increased as the temperature increased [11,12]. The rate of the strength development of concrete was affected by the W/C ratio as well as the cement type and temperature [14,15]. The mechanical properties of air-entrained concrete at elevated temperatures were studied [16]. The air entrainment had an adverse effect on the compressive strength of concrete at elevated temperatures. 

The mechanical properties of concrete can be predicted through fitting analyses of constitutive models. Hognestad et al. developed the first polynomial constitutive model for concrete, which was also one of the earliest constitutive models [17]. This model was a dimensionless full curve equation that was unified for the ascending and descending parts of the stress–strain curve. The formulation of this model provided a reference for the constitutive models developed later. To improve the accuracy of the fitting results and meet the needs of engineering, a dimensionless piecewise constitutive model was developed in which the ascending segment was a parabola and the descending segment was a straight line [18]. To precisely fit the constitutive curve of concrete under compression, the ascending curve was appropriate to be fitted by a polynomial equation and the descending curve was fitted by a rational fraction equation [19,20]. Relationships were proposed to capture the changes in the mechanical properties of concrete resulting from temperature [12,13,21]. These properties included the compressive and tensile strength, compressive strain at peak stress, and initial modulus of elasticity of concrete [21]. 

Good amounts of data exist on the effect of temperature on the mechanical properties and stress–strain relationships of concrete. However, there are limited data for this effect on concrete at different ages. Therefore, concrete cylinders were prepared, cured, and stored under different temperature conditions to be tested under compression at different ages. The stress–strain curve, mode of failure, compressive strength, ultimate strain, and modulus of elasticity of concrete were evaluated at ages between 7 and 90 days. The experimental results were used to propose constitutive models to predict the mechanical properties of concrete under the effect of temperature. Moreover, previous constitutive models for the stress–strain relationships of concrete at normal temperatures were examined to capture these relationships under the effect of temperature.

## 2. Problem Formulation

Concrete cylinders were prepared to consider the effect of temperature conditions on the stress–strain curve, mode of failure, compressive strength, ultimate strain, and modulus of elasticity of concrete at different ages. For the relevant experiment, two concrete grades (C20 and C27) of seventy-five concrete specimens each were used in this evaluation. The specimens were prepared according to ASTM C31/C31M [22]. 

### 2.1. Materials

**Figure 1:**
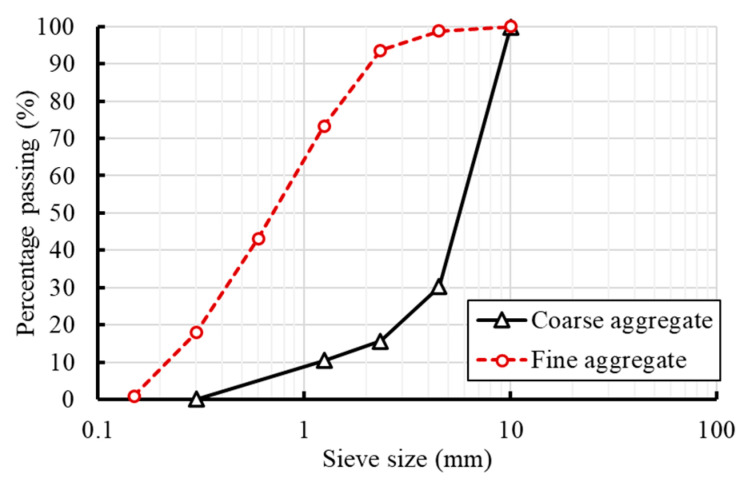
Aggregate grading curves.

To address the temperature effect alone, no admixtures were added to the concrete mix to counterbalance the alteration in workability due to temperature effects. Table 1 summarizes the concrete mix ratios. Two concrete grades were adopted in this study with different W/C ratios. Coarse aggregates with a maximum aggregate size of 10-mm continuously graded gravel aggregates were used, whereas the fine aggregate adopted river sand with a fineness modulus of 3.36. Figure 1 demonstrates the aggregate grading curves used in the preparation of concrete. The concrete mixtures were mixed using Ordinary Portland Cement type (I) manufactured by QUIKRETE and conformed to the specifications of ASTM C150 [23]. Table 2 provides the chemical composition of the used cement as provided by the manufacturer. 

### 2.2. Mixing, Fabrication, and Curing of Specimens

**Figure 2:**
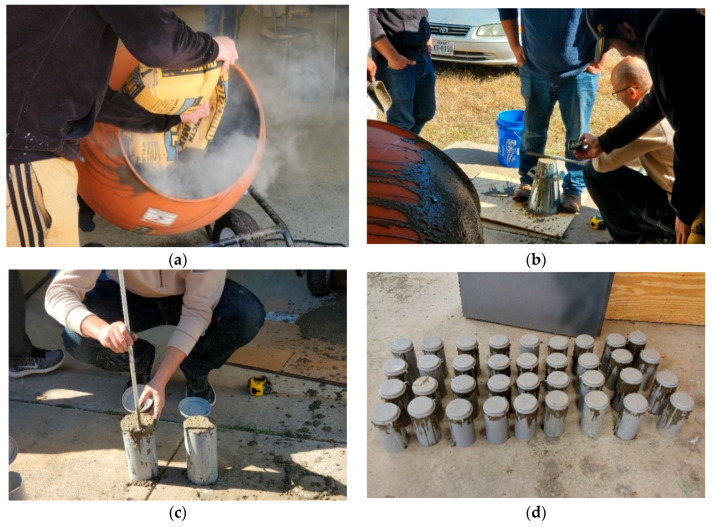
Preparation process of the concrete cylinders: (**a**) mixing concrete; (**b**) slump test; (**c**) pouring concrete; (**d**) concrete specimens in plastic molds.

To produce the concrete batches, an open pan mixer was used as shown in Figure 2a. The concrete was mixed for about 4 min until uniform color and workable consistency were achieved. All mixings were conducted at the desired temperatures. After mixing, a slump test was carried out as presented in Figure 2b and reported in Table 1. Seventy-five 100 mm × 200 mm (4 in. × 8 in.) cylinders were then cast in single-use plastic molds from each batch (C20 and C27). The specimens were prepared according to ASTM C31/C31M [22]. More details about the preparation process of the concrete cylinders are provided in Figure 2. A total of one hundred and fifty concrete cylinders were fabricated for this study. Finally, the plastic cylinders were covered to maintain the concrete water content and the concrete specimens were kept in the molds for 24 h. After removing the specimens from the plastic molds, the curing process started for 7 days at the desired temperature.

### 2.3. Detection of The Temperature Effect on the Mechanical Properties of Concrete 

**Figure 3:**
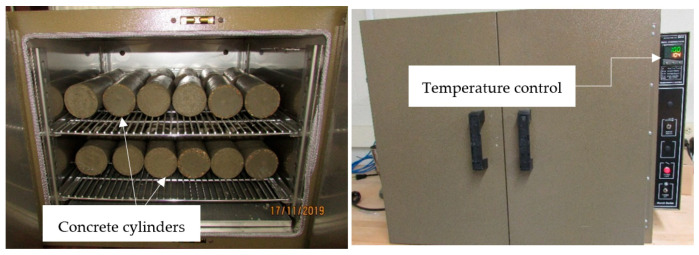
Temperature control ovens.

To thoroughly investigate the influence of temperature on the mechanical properties of hardened concrete, the specimens were cured and stored at five different temperatures; 0 °C (32 °F), 21 °C (70 °F), 40 °C (104 °F), 121 °C (250 °F), and 260 °C (500 °F). The concrete specimens were stored in temperature control ovens at the desired temperature until the day of testing as shown in Figure 3.

**Figure 4:**
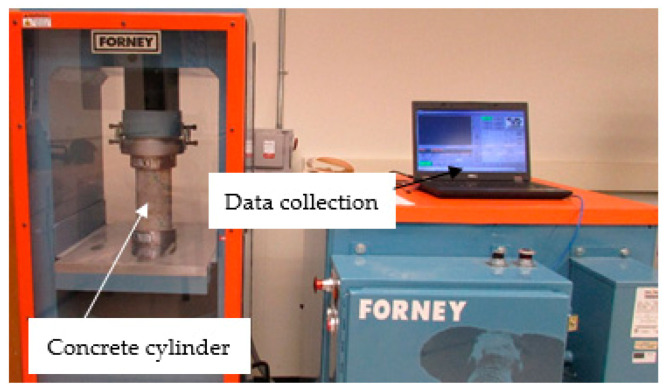
Test set-up.

To detect the mechanical properties of concrete, standardized compression tests were performed after the specimens had come to the thermal equilibrium of the normal laboratory temperature. These tests were carried out according to ASTM C39/C39M–20 [24] using a closed-loop servo-controlled standard compression machine working under a force-control with a loading rate of 9.0 kN/min (see Figure 4). The compression tests were conducted at 7, 14, 28, 56, and 90 days. Three specimens were tested for each group and the average results were adopted.

## 3. Test Results and Discussion

The mechanical properties of the tested specimens were investigated by monitoring the stress–strain relationship, mode of failure, compressive strength, ultimate strain, and modulus of elasticity as following. 

### 3.1. Stress–Strain Relationships

The stress–strain relationships of concrete are often used as input data in mathematical models. Therefore, it is crucial to evaluate the temperature effect on these relationships to explore the thermal resistance of concrete structural members. The stress–strain curve of concrete is mainly constructed of an ascending part, which ends at the compressive strength and the corresponding ultimate strain, and a descending part [25]. 

**Figure 5:**
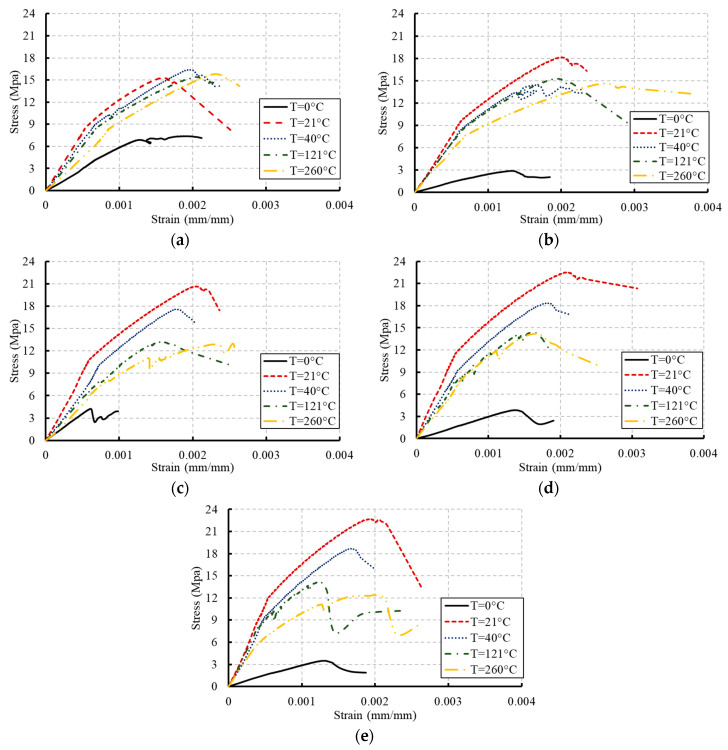
Stress–strain curves for concrete with grade C20: (**a**) at 7 days; (**b**) at 14 days; (**c**) at 28 days; (**d**) at 56 days; (**e**) at 90 days.

**Figure 6:**
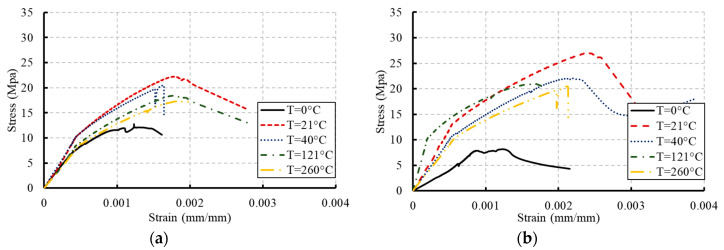

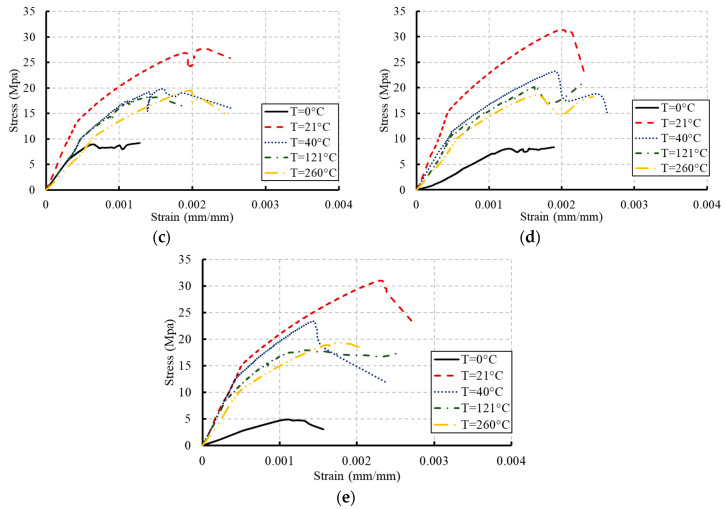
Stress–strain curves for concrete with grade C27: (**a**) at 7 days; (**b**) at 14 days; (**c**) at 28 days; (**d**) at 56 days; (**e**) at 90 days.

The behavior of the stress–strain relationships of concrete under compression is inevitably changed due to the damage caused by temperature [21]. These changes at different ages are illustrated in Figure 5 and Figure 6 for the two grades of concrete considered in this research (C20 and C27). At an early age, the strains corresponding to peak stresses had not been affected by the temperature increase. However, more reductions in these strains occurred because of freezing. With the increase in exposure to temperature, the peak stresses decreased and caused reductions in the area under the curve. The concrete started to exhibit a more brittle response by increasing the exposed temperature level [26].

The increasing rate of slopes of the linear portions of these relationships decreased through the concrete age as the temperature increased. This behavior indicated reductions in the stiffness of concrete. However, dramatic reductions were monitored in the case of freezing. These reductions are attributed to the alteration in the physical form of the hardened cement paste when the hydration proceeds at lower and faster rates in the case of freezing and high temperature, respectively [2,6,27]. Stiffness degradations and strength deteriorations occurred in concrete due to this alteration. Alternatively, the moderate temperature (21 °C) was the ideal temperature to gain the full strength of concrete over time.

**Figure 7:**
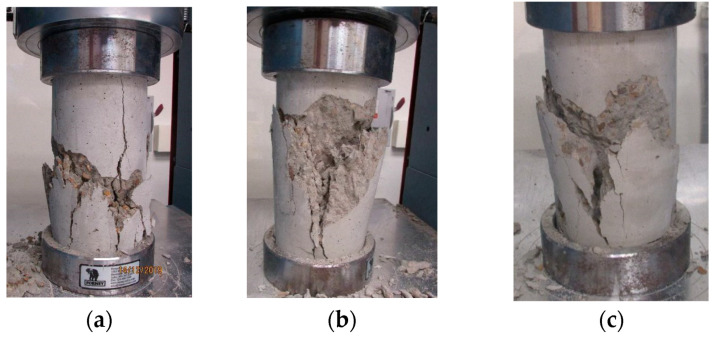

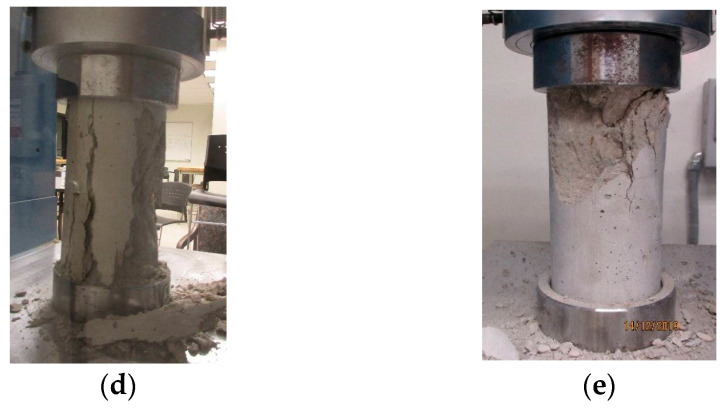
Modes of failure at age of 28 days: (**a**) T = 0 °C (well-formed cone and vertical cracks); (**b**) T = 21 °C (well-formed cone); (**c**) T = 40 °C (well-formed cone); (**d**) T = 121 °C (columnar vertical cracking); (**e**) T = 260 °C (side fractures at top).

The exposure to different temperature conditions altered the mode of failure of the tested specimens as shown in Figure 7. Three failure modes were captured for the tested cylinders. Well-formed cones and vertical cracks were the modes of failure adopted by the frozen concrete cylinders. Well-formed cones without vertical splitting were observed for the concrete cylinders at the temperatures 21 °C and 40 °C. Columnar vertical cracking was the mode of failure observed at a temperature of 121 °C. Side fractures at the top or bottom of the concrete cylinders were the mode of failure noticed for the specimens at the highest temperature (260 °C). Such failure modes were influenced by the thermal stresses associated with the temperature gradients and caused variations of damage in the microstructure of concrete [28].

### 3.2. Compressive Strength

**Figure 8:**
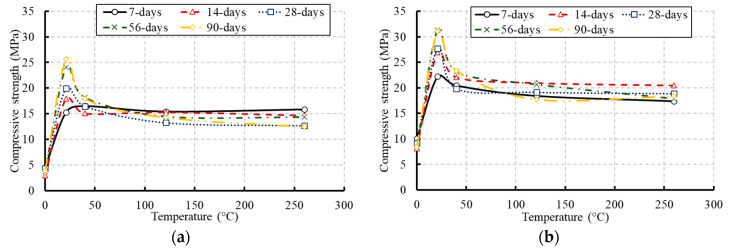
Effect of temperature on the compressive strength of concrete at different ages: (**a**) for concrete of grade C20; (**b**) for concrete of grade C27.

**Figure 9:**
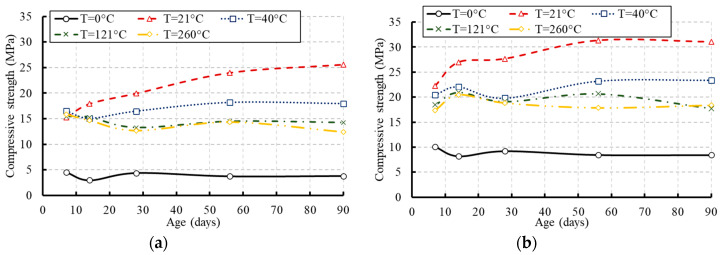
Effect of concrete age on the compressive strength at different temperatures: (**a**) for concrete of grade C20; (**b**) for concrete of grade C27.

**Figure 10:**
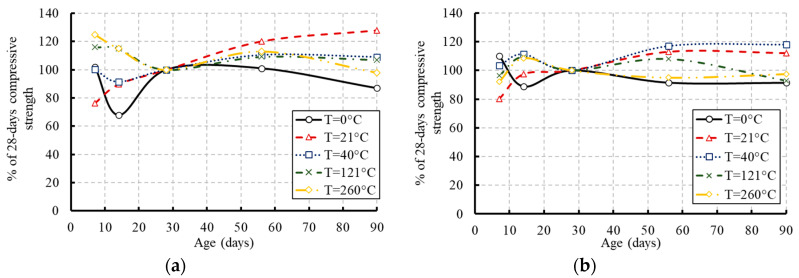
Compressive strength development of concrete as a percentage of its 28-day compressive strength: (**a**) for concrete of grade C20; (**b**) for concrete of grade C27.

**Figure 11:**
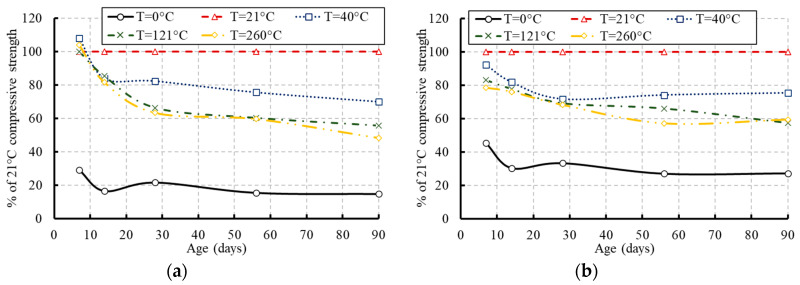
Compressive strength development of concrete as a percentage of the compressive strength at 21 °C: (**a**) for concrete of grade C20; (**b**) for concrete of grade C27.

The average compressive strength of each tested group is summarized in Table 3 and illustrated in Figure 8, Figure 9, Figure 10 and Figure 11. The presented data are the average results of three concrete cylinders tested for each group at a certain age with a standard deviation range of 2.79% to 3.15%. Figure 8 presents the influence of the temperature on the compressive strength of concrete at different ages. As demonstrated by the results, the compressive strength of the frozen specimens remained at low levels over their ages. No significant strength was earned over the concrete age. Keeping the concrete cylinders frozen at an early age could be very harmful and caused more damage to the concrete strength. The water in the concrete mix was frozen and the final product would be irreparably damaged because the temperature dipped too low [29]. Therefore, it is particularly important to protect concrete against freezing at an early age if possible, to complete the hydration process and gain most of the strength. Otherwise, most of the compressive strength was achieved at the early age of the specimens exposed to high levels of temperature. The hydration process of cement was highly affected by temperature where the reaction became faster as the temperature increased [30]. Hydration at a faster rate made the physical form of the hardened cement paste to be less well-structured and more porous. Figure 9 and Figure 10 demonstrate the effect of the concrete age on the compressive strength at different temperatures and the development in the compressive strength of concrete as a percentage of its 28-day strength, respectively. These figures confirm reductions in the compressive strength of the tested specimens at later ages due to the elevated temperatures.

The specimens exposed to the moderate temperature of 21 °C achieved only 75% of their 28-day compressive strength at the early age of concrete and further strength was achieved over the concrete age (see Figure 9). The development of the compressive strength as a percentage of the strength at an equivalent age of concrete cured and stored at 21 °C is presented in Figure 11. The highest strength was achieved by the specimens exposed to the temperature of 21 °C.

**Figure 12:**
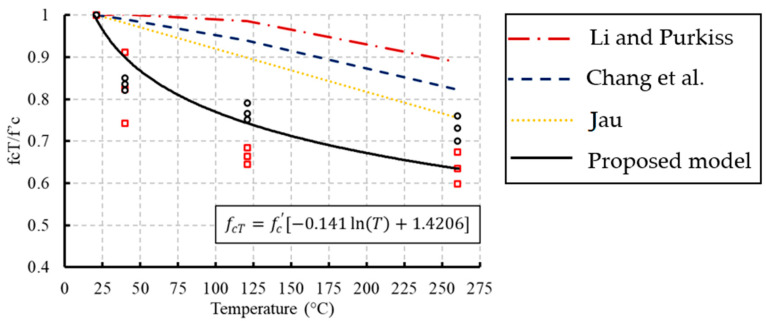
Comparisons between compressive strength at different temperatures with experimental data.

Previous constitutive models from the literature to predict the compressive strength of concrete under the effect of temperature are summarized in Table 4. The experimental data were used to validate these models and to propose a new relationship. Regression analyses were conducted on the experimental results to propose this relationship as expressed in Equation (1). The analysis was conducted on the temperature range of 21 to 260 °C. The variation of the compressive strength due to temperature compared with the proposed and previous models is shown in Figure 12.

(1) $$f_{c}^{\prime }=f_{cT}\left[-0.141\ln\left(T\right)+1.4206\right]\;\;\;20\;{}^{\circ}C\leq T\leq 260\;{}^{\circ}C$$

Based on the experimental data and the proposed model, concrete lost 10–20% of its original compressive strength when heated to 100 °C and 30–40% at 260 °C. A previous study confirmed 10–20% loss of the original compressive strength when heated to 300 °C and 60–75% at 600 °C [13]. Moreover, the proposed model fits well with the test results with a goodness of fit (R^2^) equal to 0.94. The experimental results and the proposed model indicated a non-linear relationship between the compressive strength and temperature. However, the previous models gave more conservative values and showed linear relationships. More experimental tests are required at a variety of temperatures lower than 21 °C to develop another constitutive model to consider the effect of low temperatures and freezing on the compressive strength of concrete.

### 3.3. Ultimate Strain Corresponding to the Compressive Strength

**Figure 13:**
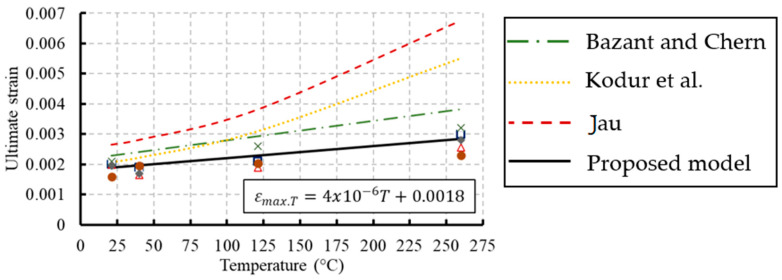
Comparison between ultimate strains at different temperatures with experimental data.

The ultimate strain corresponding to the maximum stress increased as the temperature increased as demonstrated in Figure 13. Previous models were developed to predict this strain at elevated temperatures as summarized in Table 5. Moreover, regression analyses were conducted on the experimental results to develop a new relationship to predict the ultimate strain as expressed in Equation (2). The analysis was conducted on the temperature range from 21 to 260 °C. The proposed formula represented a linear relationship between the ultimate strain and temperature and fits well with the test results with R^2^ equal to 0.91.

(2) $$\varepsilon_{max.T}=4\times 10^{-6}T+0.0018\;\;\;20\;{}^{\circ}C\leq T\leq 260\;{}^{\circ}C$$

### 3.4. Modulus of Elasticity

**Figure 14:**
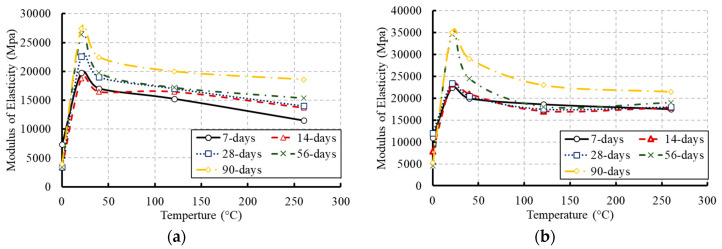
Effect of temperature on the modulus of elasticity of concrete over time: (**a**) for concrete of grade C20; (**b**) for concrete of grade C27.

The slope of the linear portion of the stress–strain relationship for concrete is defined as the initial tangent modulus of elasticity which was considered in this study. The modulus of elasticity is dependent mainly on the W/C ratio in the mixture, the age of concrete, and temperature [36]. The variation in the elastic modulus due to temperature at different ages is illustrated in Figure 14 and summarized in Table 6. The results indicated that there was a highly negative correlation between the modulus of elasticity of concrete and temperature. The modulus of elasticity decreased as the temperature increased. At elevated temperature, the disintegration of hydrated cement products and breakage of bonds in the microstructure of cement paste reduced the elastic modulus and the extent of this reduction depended on the moisture loss, high temperature, and type of aggregate [10]. However, significant reductions in these values occurred for the frozen concrete specimens. A new constitutive model for the modulus of elasticity of concrete under the effect of temperature was proposed using the experimental data as expressed in Equation (3). The developed model was fitted well with most of the experimental results with R^2^ equal to 0.97.

(3) $$E_{cT}=E_{c}\left[-0.125\ln\left(T\right)+1.359\right]\;\;\;20\;{}^{\circ}C\leq T\leq 260\;{}^{\circ}C$$

The modulus of elasticity of concrete can be predicted using empirical methods provided in the current standards. These methods depend on the compressive strength of concrete. According to ACI 318-14 [37], the modulus of elasticity is calculated based on the 28-day compressive strength (*f’_c_*) using the following Equation (4):

(4) $$E_{c}=4700\sqrt{f_{c}^{\prime }}$$

**Figure 15:**
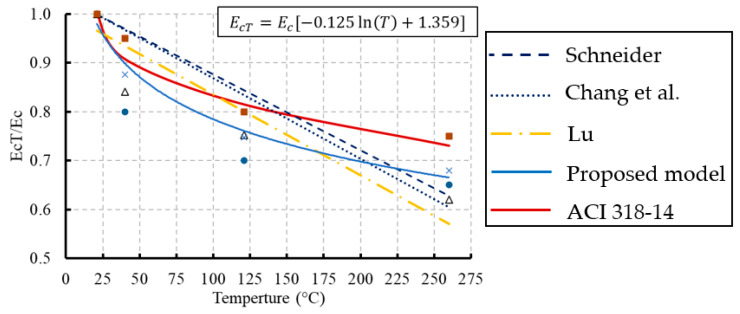
Comparisons between modulus of elasticity developed by ACI 318-14 and the 28-day experimental results: (**a**) for concrete of grade C20; (**b**) for concrete of grade C27.

To consider the effect of temperature in the ACI 318-14 equation, the compressive strength affected by temperature and predicted by Equation (1) was used. Comparisons were conducted between the modulus of elasticity experimentally measured and analytically predicted as presented in Figure 15. The analytical results were obtained by the new proposed model, the previous models (Table 7), and the ACI 318-14 equation. The previous analytical models were fitted in a linear relationship to consider the effect of the temperature. However, the experimental results and the new model showed a non-linear relationship for the modulus of elasticity because of temperature. Additionally, the modulus of elasticity of concrete predicted by the ACI 318-14 equation was matching well with the experimental results.

## 4. Previous Constitutive Models for the Stress–Strain Relationships

Previous constitutive models were proposed to trace the compressive stress–strain relationship of concrete at normal temperatures. The constitutive models proposed by Shah et al. [40] and Kent and park [41] were examined in this study to represent the compressive stress–strain relationship of concrete under the effect of temperature (see Table 8). The newly affected mechanical properties of concrete (compressive strength, ultimate strain, and modulus of elasticity) due to temperature were used in these models. 

Where *f_c_* is the stress value at any strain *ε_c_*, *f_c_^′^* and *ε_co_* are the maximum stress and the corresponding ultimate strain obtained from the proposed models (Equations (1) and (2)), respectively. The parameters A and K are used to determine the shape of the curve in the ascending and descending parts, respectively. The modulus of elasticity (*E_c_*) of concrete is obtained based on the proposed model for concrete under the effect of temperature (Equation (3)). *ε_50u_* is the strain corresponding to 50% of the maximum concrete strength.

**Figure 16:**
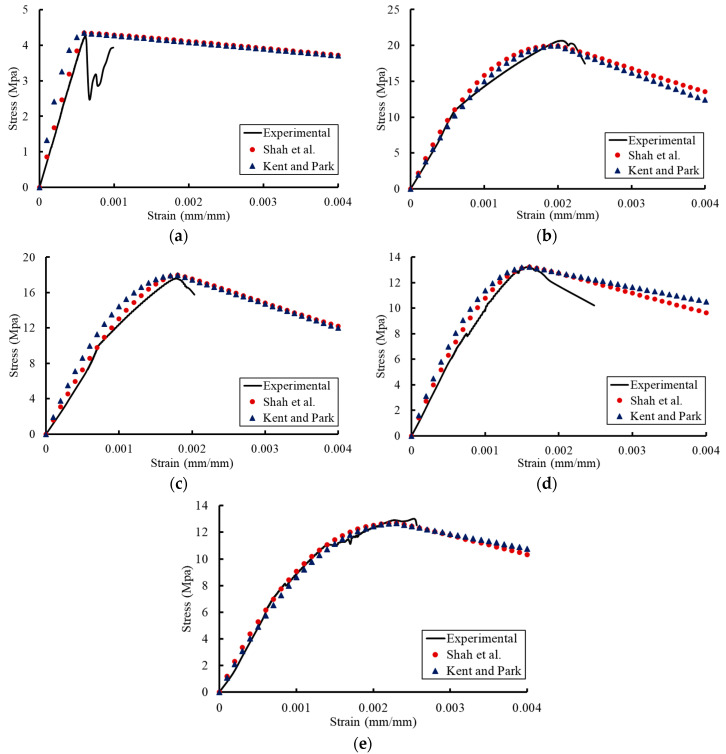
Comparisons between the Shah et al. model and the experimental results for concrete of grade C20: (**a**) T = 0 °C; (**b**) T = 21 °C; (**c**) T = 40 °C; (**d**) T = 121 °C; (**e**) T = 260 °C.

**Figure 17:**
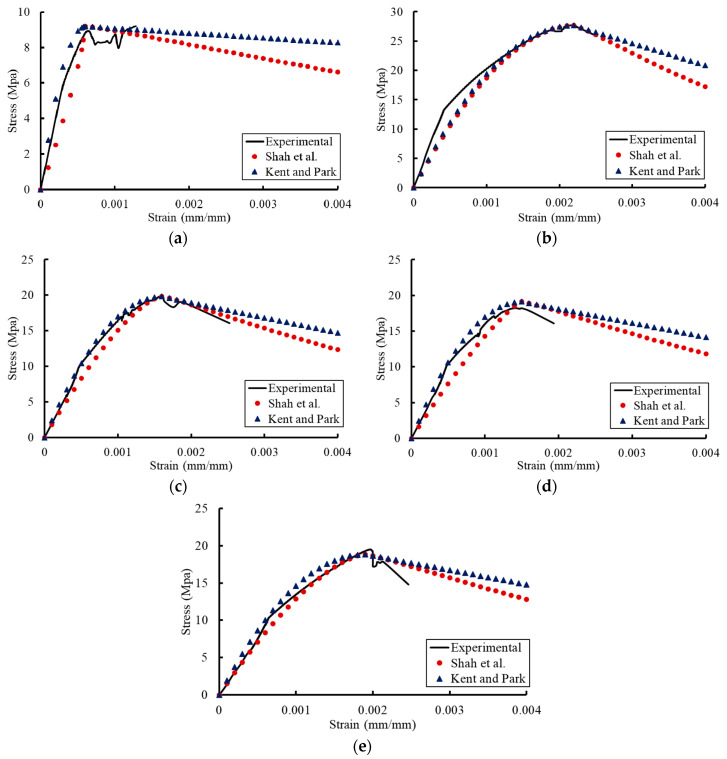
Comparisons between the Shah et al. model and the experimental results for concrete of grade C27: (**a**) T = 0 °C; (**b**) T = 21 °C; (**c**) T = 40 °C; (**d**) T = 121 °C; (**e**) T = 260 °C.

Figure 16 and Figure 17 provide comparisons between the previous constitutive models after considering the temperature effect and the experimental results. The previous models presented analytical results which had good agreements with the experimental results. These models reflected the affected compression stress–strain relationships due to temperature by using the mechanical properties of concrete predicted by the proposed models. Therefore, it can be concluded that the previous constitutive models for stress–strain relationships of concrete at normal temperatures can be used to capture these relationships under the effect of temperature by using the compressive strength, ultimate strain, and modulus of elasticity that were affected by temperature.

## 5. Conclusions

In this study, an overview of the temperature effect on concrete is experimentally provided. Concrete cylinders were prepared, cured, and stored under different temperature conditions to be tested under compression. The stress–strain curve, mode of failure, compressive strength, ultimate strain, and modulus of elasticity of concrete were evaluated at ages between 7 and 90 days. The experimental results were used to validate previous constitutive models and to develop new models to predict the mechanical properties of concrete under the effect of temperature.

1. The previous constitutive models for stress–strain relationships of concrete at normal temperatures can be used to capture these relationships under the effect of temperature by using the compressive strength, ultimate strain, and modulus of elasticity affected by temperature and developed in this study.
2. The effect of temperature on the modulus of elasticity of concrete can be considered in the ACI 318-14 equation by using the compressive strength affected by temperature.
3. The increasing rate of slopes of the linear portions of the stress–strain relationships of concrete decreased through the concrete age as the temperature increased. This behavior indicated a reduction in the stiffness of concrete. However, dramatic reductions were monitored in the case of freezing.
4. The exposure to different temperature conditions altered the mode of failure of the tested specimens. A well-formed cone without vertical splitting was the mode of failure observed for the concrete cylinders at 21 °C and 40 °C. Columnar vertical cracking was the mode of failure observed at a temperature of 121 °C. Side fractures at the top or bottom of the concrete cylinders were the mode of failure noticed for the specimens at the highest temperature (260 °C).
5. Based on the experimental data and the newly proposed model, concrete lost 10–20% of its original compressive strength when heated to 100 °C and 30–40% at 260 °C.
6. The compressive strength of the frozen specimens remained at low levels over their ages. No significant strength was earned as the concrete aged. Keeping the concrete cylinders frozen at an early age could be very harmful and caused more damage to the concrete strength. Therefore, it is particularly important to protect concrete from freezing at an early age if possible, to complete the hydration process and gain most of the strength.
7. Most of the compressive strength was gained at the early age of concrete due to the exposure to elevated temperature. However, reductions in the strength occurred at later ages.
8. The specimens exposed to the moderate temperature of 21 °C achieved only 75% of their 28-day compressive strength at the early age of concrete and further strength was achieved as the concrete age increased.

## Figures and Tables

**Table 1:** The concrete mix ratios.

Concrete Grade	Design Strength (MPa)	Aggregate Size * (mm)	Slump (mm)	W/C	Gravel (kg/m^3^)	Sand (kg/m^3^)	Portland Cement (kg/m^3^)	Fly Ash (kg/m^3^)
C20	20	10	127.20	0.55	1067.9	830.6	334.6	30
C27	27	10	82.55	0.45	1067.9	830.6	334.6	30

* Maximum aggregate size.

**Table 2:** Chemical composition of the used cement.

Constituent	% by Weight *
Lime (CaO)	65.00
Silica (SiO_2_)	21.00
Alumina (Al_2_O_3_)	5.60
Iron Oxide (Fe_2_O_3_)	3.80
Magnesia (MgO)	2.10
Sulphur Trioxide (SO_3_)	2.22
Loss of Ignition	0.65
Lime saturation factor	0.90

* Provided by the manufacturer (QUIKRETE Companies, Atlanta, Georgia).

**Table 3:** Compressive strength of concrete (MPa).

Time	Temperature	0 °C	21 °C	40 °C	121 °C	260 °C
7-day	C20	4.47	15.27	16.49	15.42	15.83
	C27	10.08	22.21	20.46	18.44	17.40
14-day	C20	2.97	17.93	15.05	15.29	14.64
	C27	8.17	26.96	22.06	20.95	20.47
28-day	C20	4.35	19.98	16.45	13.25	12.67
	C27	9.19	27.68	19.81	19.12	18.85
56-day	C20	3.73	24.01	18.16	14.47	14.33
	C27	8.43	31.34	23.19	20.66	17.89
90-day	C20	3.79	25.61	17.93	14.24	12.40
	C27	8.41	31.03	23.37	17.74	18.40

**Table 4:** Previous compressive strength models of concrete at high temperatures.

Reference	Compressive Strength Models
Li and Purkiss [31]	$f_{cT}=f_{c}^{\prime }\left[0.00165{\left(\frac{T}{100}\right)}^{3}-0.03{\left(\frac{T}{100}\right)}^{2}+0.025\left(\frac{T}{100}\right)+1.002\right]$
Jau [32]	$f_{cT}=f_{c}^{\prime }\left(1-0.001T\right)$
Chang et al. [33]	$f_{cT}=f_{c}^{\prime }\left[1.008+\frac{T}{450\mathrm{lna}?\left(\frac{T}{5800}\right)}\right]$

**Table 5:** Previous ultimate strain models at elevated temperatures.

Reference	Ultimate Strain Models
Bazant and Chern [34]	$\varepsilon_{max.T}=0.0000064T+0.0021$
Lie [35]	$\varepsilon_{max.T}=0.0025+\left(6.0T+0.04T^{2}\right)\times 10^{-6}$
Kodur et al. [10]	$\varepsilon_{max.T}=0.0018+\left(6.7f_{c}^{\prime }+6.0T+0.03T^{2}\right)\times 10^{-6}$

**Table 6:** Modulus of elasticity of concrete (MPa).

Time	Temperature	0 °C	21 °C	40 °C	121 °C	260 °C
7-day	C20	7319.28	19,762.05	17,036.25	15,236.05	11,474.74
	C27	11,821.12	22,456.88	20,249.47	18,639.21	17,542.78
14-day	C20	3433.34	18,821.00	16,468.38	16,468.38	13,701.69
	C27	7904.82	23,249.47	19,762.05	17,067.23	17,884.21
28-day	C20	3426.62	22,585.20	19,937.14	17,242.92	14,161.26
	C27	12,000.25	23,400.65	20,500.1	17,500.02	18,201.58
56-day	C20	3423.95	26,349.40	19,762.05	17,207.77	15,370.48
	C27	4526.056	34,670.26	24,467.30	18,068.16	19,077.6
90-day	C20	3952.41	27,447.29	22,456.88	20,702.56	18,599.58
	C27	5269.88	35,000.31	29,061.84	23,231.5	21,480.49

**Table 7:** Previous modulus of elasticity models at elevated temperatures.

Reference	Modulus of Elasticity Models
Schneider [38]	$E_{cT}=\left(-0.001552T+1.03104\right)E_{c}$
Lu (reported in ref. [39])	$E_{cT}=\left(1-0.0015T\right)E_{c}$
Chang et al. [33]	$E_{cT}=\left(-0.00165T+1.033\right)E_{c}$

**Table 8:** Previous models for stress–strain relationships at normal temperatures.

Reference	Stress–Strain Models	
Shah et al. [40]	$f_{c}=f_{c}^{\prime }\left[1-{\left(1-\frac{\varepsilon_{c}}{\varepsilon_{co}}\right)}^{A}\right]$	Ascending part
	$f_{c}=f_{c\;}^{\prime }e^{-k{\left(\varepsilon_{c}-\varepsilon_{co}\right)}^{1.15}}$	Descending part
	$k=0.17\;f_{c}^{\prime }\;\;\;\;\;\;\;\;\;\;\;\;\;\;\;\;\;\;\;\;\;\;\;\;\;\;\;\;\;\;\;\;$ $A=E_{c}\frac{\varepsilon_{co}}{f_{c}^{\prime }}$	
Kent and Park [41]	$f_{c}=f_{c}^{\prime }\left[\left(\frac{2\varepsilon_{c}}{\varepsilon_{co}}\right)-{\left(\frac{\varepsilon_{c}}{\varepsilon_{co}}\right)}^{2}\right]$	Ascending part
	$f_{c}=f_{c}^{\prime }\left[1-\frac{0.5}{\varepsilon_{50u}-\varepsilon_{co}}\left(\varepsilon_{c}-\varepsilon_{co}\right)\right]$	Descending part
	$\varepsilon_{50u}=\frac{3+0.29f_{c}^{\prime }}{145f_{c}^{\prime }-1000}$	
